# Editorial: Multimodal and auto-regulation monitoring in neuro-ICU

**DOI:** 10.3389/fneur.2023.1227237

**Published:** 2023-07-14

**Authors:** Rohan Sharma, Jeffrey Peel, Nidhi Kapoor, William D. Freeman

**Affiliations:** ^1^Mayo Clinic Florida, Jacksonville, FL, United States; ^2^Baptist Medical Center Jacksonville, Jacksonville, FL, United States

**Keywords:** Multimodal Monitoring, EEG, NIRS (near infrared reflectance spectroscopy), TCD (transcranial doppler sonography), PbO2

“*Hypotheses are nets, only he who casts will catch*.” – NOVALIS.

Is Multimodal Monitoring (MMM) still primarily a research tool? Do most NeuroICUs now use some form of MMM in their practice to call themselves MMM-capable? What is the ground truth against which MMM is based scientifically? *Veridical science* is the ability to find truth amidst reality^1^. To find truth, one must be able to prove or disprove a hypothesis as true or false. Popperian falsifiability (1) is key to the scientific method since without the ability to prove something false, how would one ever find the “truth”? Veridical science uses PCS (predictability, computability, and stability) to determine truth. Why is this important in the NeuroICU with MMM? The intensive care unit is a big data environment given its *V's* (high *V*olume, high *V*elocity, high *V*ariety) of data. Therefore, creation of predictable (e.g., provable), computable, and stable systems are critical to discover physiologic truths, and later test interventions against them.

## The promise of MMM

MMM research is promising with discoveries providing insights about optimal Cerebral Perfusion Pressure (CPP_opt_) in abnormal brain physiology to individualize care for brain injured patients. Patients admitted to NeuroICUs often have severe neurological injury. The job of intensivists is not only to manage the direct complications from the primary neurological damage but also to prevent and limit the secondary neurological injury that ensues following the initial insult. The secondary damage can result from various mechanisms including ischemia, excito-toxicity, neuro-inflammation, and cellular apoptosis. Predicting this secondary damage is still an elusive task and requires a multifaceted approach.

**Figure 1:**
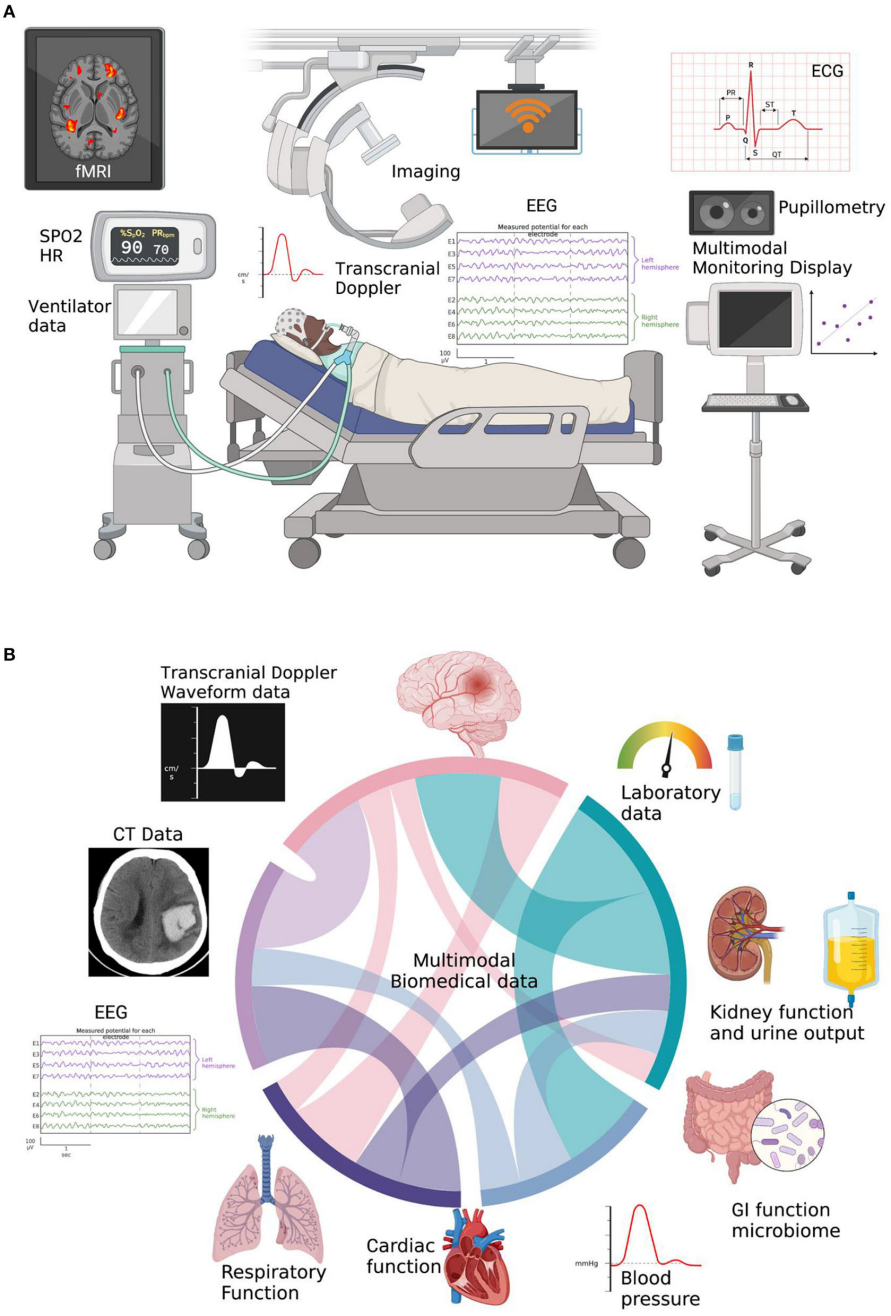
**(A)** Current state of MultiModal Monitoring (MMM) data in the NSICU, which currently is all separate and hard to time-sync and label without a MMM-platform to make scientific inferences. Created with BioRender. **(B)** Cord diagram showing integrated multimodal biomedical information utilizing multiparameter brain data as well as other ICU physiologic data. Created with BioRender.

MMM is promising and has potential to improve patient outcomes. MMM also has its pitfalls and practicalities (Figure 1A) such as getting all the machines in the ICU to speak to one another and be time synchronized and labeled with clinical events in one MMM platform. Unlike the traditional approach of monitoring of single variable such as intracranial pressure, cerebral blood flow or oxygenation, MMM aims at using the amalgamation of data from different modalities to individualize care for each patient to optimize outcomes (Figure 1B). Newer modalities and protocols are currently being explored to add to the existing armamentarium of intensivists.

## Practicalities in MMM

Within this issue of *Frontiers*, Kazazian et al. presented a multimodal imaging protocol as a diagnostic and prognostic tool for secondary brain injury. Their protocol aims at utilizing functional Magnetic Resonance Imaging (fMRI), electroencephalography (EEG), and functional Near Infrared Spectroscopy (fNIRS) to measure and map the brain activity of acute critically ill patients. Their ongoing analysis may lead to the development of a useful clinical tool in future. While the results are certainly promising, not all NSICU's are resourced equally and many cannot obtain fMRI much less fNIRS practically speaking -either due to resources, costs, or other logistics.

In contrast, Zhang et al. explored the relation between the two commonly used ICU modalities i.e. invasive and non-invasive arterial blood pressure monitoring and found these to have good agreement in evaluating dynamic cerebral autoregulation with recordings reaching 15 min. However, their analysis did reveal that phase shift values were higher with the non-invasive techniques. This analysis allows the clinician to utilize non-invasive techniques for cerebral auto-regulation monitoring in appropriate settings, thereby reducing the risks and side effects of invasive monitoring in selected patients.

EEG monitoring is becoming routine in NeuroICUs but subtleties and more widespread utility remain challenges, as well as indications beyond monitoring for seizures and status epilepticus. ICU EEG has been found to be useful tool in prediction of vasospasm/delayed cerebral ischemia in subarachnoid hemorrhage patients as well as in post-code patients for prognostication. Xu et al. studied the use of processed multiparameter EEG to guide anesthesia during carotid endarterectomy to mitigate postoperative delirium. They also utilized transcranial doppler (TCD) and NIRS during the surgery to monitor cerebral perfusion. This study highlighted the use of MMM beyond the ICU to prevent neurological injury. Greve et al. also analyzed the impact of age and positioning on EEG parameters in infants and children. This analysis further emphasized the utility of looking beyond the general protocols and a need for personalized care based on individual specific factors.

Coppalini et al. presented preliminary data about improvement in cerebral oxygenation pressure and perfusion with use of inotropes using invasive brain oxygen pressure (PbtO2) catheter. Although, these findings may seem intuitive, especially in patients with decrease tissue oxygenation, all patients do not linearly respond to this treatment. This suggests the necessity of larger studies to guide patient selection for inotrope therapy based on invasive and non-invasive data.

MMM has generated interest of physicians and researchers around the world, and there remain practical challenges in rural and underserved areas of the globe having access to these often-expensive MMM devices, probes, and platforms. Vitt at al. have presented a thorough review of physiology of MMM parameters to aid the understanding of this ever-evolving and expanding field. Sharma et al. presented practical recommendations for the use of MMM to assist physicians in making decisions about the choice of modality and the nuances of interpretation. Despite the potential benefits of MMM, its implementation in the NSICU can be challenging. Firstly, it requires specialized equipment and trained personnel, which may not be available in all centers. Secondly, the interpretation of the data requires expertise in multiple modalities, which may be a barrier to its use. Thirdly, MMM can generate large amounts of data, which can be overwhelming for clinicians and databases alike. Thus, to overcome these challenges, we suggest using a team science or multidisciplinary approach. MMM should be ideally implemented as part of a comprehensive management protocol that involves multiple clinicians (neuro/neurosurgeons, intensivists, nursing staff) and a data science platform that provides the PCS components centered in veridical science. More funding for prospective randomized MMM data science will help answer hypotheses like the ongoing BOOST-3 trial for traumatic brain injury (TBI) ^2^, and the National Institutes for Health (NIH) CHoRUS BRIDGE2AI funding^3^. This issue is dedicated to a deeper understanding of existing knowledge of MMM articles, with the hopes of guiding future research and aiding current clinical practice to serve our neuro critically ill patients. It's time to shine a light on neurocritical care illnesses using MMM within the resources available, and above all, it must be firmly rooted in the scientific method to determine truth versus reality.

## Author contributions

All authors listed have made a substantial, direct, and intellectual contribution to the work and approved it for publication.

## Footnotes

^1^ https://www.pnas.org/doi/10.1073/pnas.1901326117

^2^ https://clinicaltrials.gov/ct2/show/NCT03754114

^3^ https://reporter.nih.gov/project-details/10472824
